# Subacute Combined Degeneration of the Cervical Spine Secondary to Inhaled Nitrous-Oxide-Induced Cobalamin Deficiency

**DOI:** 10.7759/cureus.21214

**Published:** 2022-01-13

**Authors:** Larissa Check, Nardine Abdelsayed, Gabriela Figueroa, Aditya Ragunathan, Mohamed Faris

**Affiliations:** 1 Internal Medicine, Grand Strand Medical Center, Myrtle Beach, USA

**Keywords:** nitrous oxide myelopathy, rare cause of vitamin b12 deficiency, toxic neuropathy, neuropathy, subacute combined degeneration

## Abstract

Nitrous oxide is clinically used as an inhaled anesthetic in surgical and dental procedures. It is also used as an inhaled recreational drug and can be incredibly addictive. It tends to irreversibly oxidize cobalamin (Vitamin B12), rendering it inactive as a coenzyme in the production of methionine. Methionine is required in myelin sheath phospholipid production, and thus overuse of this anesthetic can affect myelin formation. Furthermore, other substrates that require this coenzyme (such as methylmalonate and propionate) accumulate and get incorporated in the myelin sheath, resulting in subacute combined degeneration of the spinal cord. We present a case of a young, avid hunter with a history of polysubstance use to include inhaled nitrous-oxide abuse, prior cocaine use, current marijuana use, and tobacco abuse, who presented with ascending paresthesias without appreciable motor dysfunction. Initial labs showed isolated macrocytosis without anemia in the setting of low vitamin B12 levels. Relevant studies showed elevated methylmalonic acid, normal anti-parietal cell, and anti-intrinsic factor antibodies. Heavy metals screens were negative for high levels of lead, iron, copper, or zinc. Cervical spine MRI demonstrated dorsal cord signal abnormalities without enhancement, in a pattern consistent with vitamin B12 deficiency. The patient was diagnosed with subacute combined degenerative disease secondary to depleted vitamin B12 as a result of recreational inhaled nitrous-oxide abuse. After cessation of nitrous oxide abuse, in addition to three months of B12 replacement, he reported complete resolution of symptoms.

## Introduction

Nitrous oxide is an inert and colorless gas often used as an anesthetic in clinical dentistry [1]. The mechanism of action for its anesthetic properties remains largely unknown. Its role as an anxiolytic and as a potent vasodilator has also been used historically to great benefit. However, its use has not been limited to appropriate medical and clinical practice only [2]. Nitrous oxide has been used for recreational purposes for over 200 years [1]. Commonly known as laughing gas, its use has been linked to euphoric effects and diminished perception of pain. As an analgesic, it acts as an N-methyl-D-aspartate (NMDA) antagonist and has also been hypothesized to activate noradrenergic motor neurons thereby releasing opioid peptides [2]. The resulting effect is a decrease in the perception of pain, euphoria, and depersonalization. Nitrous oxide abuse can result in minimal adverse effects for some users and asphyxia-related deaths for other users [1,2]. Heavy users, who inhale NO consistently over a period of weeks become prone to myelopathy and neuropathy, which can present as paresthesia, tingling, weakness, gait disturbance, and loss of proprioception [3].

## Case presentation

We present a case of a 29-year-old male with a past medical history of polysubstance abuse who presented with a chief complaint of progressive bilateral ascending paresthesia of the upper and lower extremities without motor dysfunction. The paresthesia of the hands and feet started 10 days prior to admission. The patient denied focal weakness and his complaints were purely sensory. He did report posterior neck pain that started a couple of days after numbness and tingling in his fingertips and feet. The pain did not radiate toward the neck or down the back. The patient worked as a cook in a restaurant and his symptoms had been interfering with his job. He denied any recent tick bites. He was an avid hunter and loved to eat “game meat.” The patient denied a change in diet, use of metformin and had no history of bariatric surgery. He also had no family history of type 1 diabetes, autoimmune disorders, multiple sclerosis, or amyotrophic lateral sclerosis. Furthermore, he also denied having had a recent upper respiratory infection, viral illness, vaccination, or gastrointestinal symptoms. The patient admitted using cocaine and heroin in the past but has not used these drugs in the past two years. He did admit to recreational use of inhaled nitrous oxide during this time.

**Figure 1:**
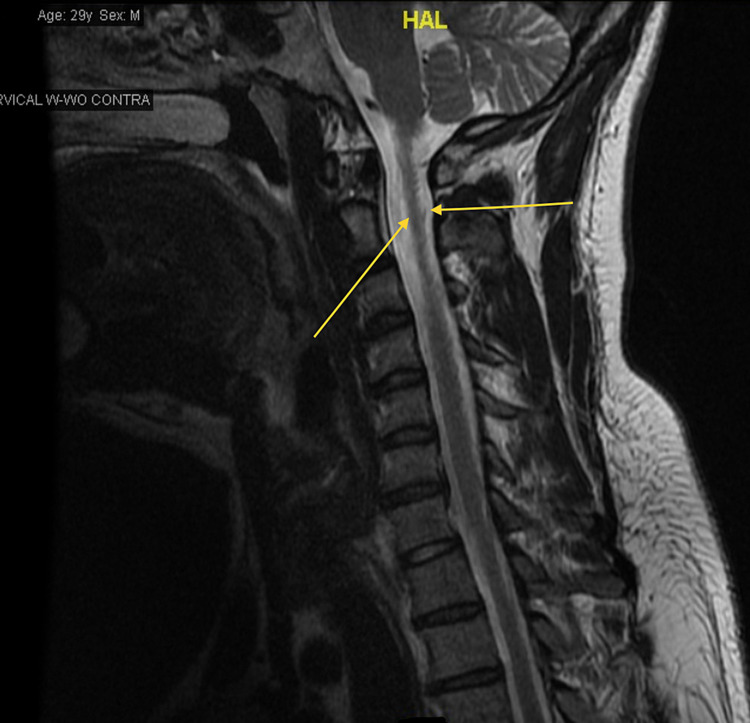
Multiplanar multi-sequence magnetic resonance imaging (MRI) of the cervical spine showing that the cervical cord demonstrated symmetric posterior medial signal abnormalities in a pattern consistent with vitamin B12 deficiency (yellow arrows). No other pathologic enhancement was seen within the cervical cord, meninges, or vertebral bodies.

Throughout his hospital stay, his vitals were within normal limits. On physical examination, strength was preserved in all four extremities. His gait was observed to be without overt abnormalities, however, he endorsed a lack of awareness of his limbs’ movements in space. There were no signs of hyperpigmentation over the over knuckles or MTP joints, Romberg's sign was negative and no ataxia in gait. Laboratory findings showed hemoglobin (Hgb) A1c of 5.1 (3.8%-5.6%), calcium 9.5 (8.4-10.2 mg/dL), C-reactive protein <0.50 (0-0.99 mg/dL), Vitamin B12 166 (239-931 pg/mL), folate 11.0 (2.76-20.0 ng/mL), thyroid-stimulating hormone (TSH) 5.79 (0.465-4.68 µIU/mL), and free T4 0.88 (0.78-2.19 ng/dL). Complete blood count (CBC) showed Hgb 15.4 (14.0-16.4 g/dL), Hct 45.6 (40.0%-47.2%), mean corpuscular volume (MCV) 101.8 (81.8-94.6 fL), and erythrocyte sedimentation rate (ESR) 1.0 (0-15 mm/hr). Multiplanar multi-sequence magnetic resonance imaging (MRI) of the cervical spine was performed both before and after the administration of intravenous contrast. The cervical spine MRI revealed dorsal cord signal abnormalities without enhancement in a pattern consistent with vitamin B12 deficiency (Figure 1).

We initiated therapy with 1 gram of Vitamin B12 injection. The patient was instructed to continue B12 replacement with 1 gram, once a week for three weeks and thereafter, 1 gram once a month. Neurology consulted and agreed with the diagnosis, exam findings of subacute combined degeneration. Further workup showed elevated methylmalonic acid, normal anti-parietal cell, and anti-intrinsic factor antibodies. Heavy metals screens were negative for high levels of lead, iron, copper, and zinc. Given the patient’s non-vegetarian nutrition, negative autoimmune workup, it was determined that the patient’s B12 deficiency and megaloblastic anemia were the results of heavy nitrous oxide. Upon discharge, he was counseled on tobacco cessation in addition to nitrous oxide cessation. He was also seen in the outpatient clinic and reported improvement in his symptoms with B12 replacements and complete resolution of his symptoms three months later.

## Discussion

Vitamin B12 is an essential nutrient and cofactor to methylmalonyl-CoA mutase, the enzyme required for the biochemical conversion of methylmalonyl-CoA to succinyl-CoA in the mitochondria as well as the conversion of 5-methyltetrahydrofolate to tetrahydrofolate, two important reactions for healthy neurologic function, DNA synthesis, and hematopoiesis [4]. Deficiency in vitamin B12 leads to an accumulation of methylmalonic acid (MMA), manifesting as hematologic abnormalities or neurologic/neuropsychiatric symptoms. Vitamin B12 is acquired mainly through dietary sources such as fish, meat, and dairy. It is absorbed in the terminal ileum alongside intrinsic factor, which is produced by the parietal cells of the stomach. Deficiency in an individual can result from either poor dietary intake or malabsorption [4]. The most common cause of vitamin B12 deficiency is malabsorption from food sources, which occurs when the release of vitamin B12 is impaired due to an outstanding factor that prevents its release from its transport protein. This process occurs in circumstances that include achlorhydria, gastritis, gastrectomy, the use of proton pump inhibitors (PPIs) or antacids. Another cause of vitamin B12 deficiency, though less common, is pernicious anemia. This disease is defined by an autoimmune process that causes chronic atrophic gastritis, leading to the destruction of parietal cells which then impairs intrinsic factor production and release into the gut followed by the inability of the body to absorb any readily available vitamin B12 [4].

Patients with vitamin B12 deficiency have impaired deoxyribonucleic acid (DNA)/ribonucleic acid (RNA) production leading to macrocytic megaloblastic anemia which may present at increased fatigue, dizziness, lightheadedness, tachycardia, and possible syncope. It may also be found on routine laboratory findings with low hemoglobin and elevated MCV. The most notable manifestation of chronic vitamin B12 deficiency is its associated neurological symptoms - numbness, paresthesias, loss of vibration and proprioception, which cumulatively lead to balance deficits and frequent falls. Some of the neurological symptoms are unfortunately irreversible. Furthermore, psychiatric manifestations may occur including depression, irritability, and a notable loss of cognition [3]. Homocysteine accumulates in vitamin B12 deficiency which tends to be associated with increased risk of coronary artery disease, cerebrovascular accidents, and peripheral artery disease. Some gastrointestinal manifestations include cheilosis, gastritis, diarrhea or constipation, and occasionally weight loss. Overall, this disorder is by no means benign and requires urgent treatment [3]. Vitamin B12 supplementation, whether it be by parenteral or intramuscular routes is essential to prevent further clinical decline.

## Conclusions

The medical management for vitamin B12 deficiency incorporates patient history, physical examination findings, and laboratory testing. Historical data should include a list of medications such as metformin and cimetidine, nutrition intake (strict vegetarianism), nitrous oxide abuse, alcohol use disorder, family and personal history of autoimmune diseases. During initial laboratory analysis, a CBC, peripheral blood smear (PBS), folate (vitamin B9), and cobalamin (vitamin B12) levels should be obtained. The CBC may reveal macrocytic anemia or isolated macrocytosis while the PBS may reveal neutrophil hypersegmentation. If the clinical and laboratory findings are concordant with vitamin B12 deficiency, additional testing for homocysteine, MMA, intrinsic factor and anti-parietal cell antibody may be warranted. It is important to note that most findings for vitamin B12 and B9 deficiencies are similar except MMA is elevated in vitamin B12 deficiency but not in vitamin B9 deficiency. Once the diagnosis of vitamin B12 has been established and optimal therapy initiated, improvement in symptoms further supports the diagnosis. Our patient reported complete resolution of his symptoms three months after vitamin B12 replacement was initiated. He also reported abstaining from nitrous oxide abuse.
